# Development of a high‐throughput dual‐stream liquid chromatography–tandem mass spectrometry method to screen for inhibitors of glutamate carboxypeptidase II

**DOI:** 10.1002/rcm.9772

**Published:** 2024-06-12

**Authors:** Nate Hoxie, Yixuan Qiu, Stephen C. Kales, Rick Schneider, Xin Hu, Anu Dalal, Stephanie L. Ford‐Scheimer, Robyn Wiseman, Takashi Tsukamoto, Huijun Wei, Barbara S. Slusher, John S. Janiszewski, Matthew D. Hall

**Affiliations:** ^1^ National Center for Advancing Translational Sciences National Institutes of Health Rockville Maryland USA; ^2^ Johns Hopkins Department of Pharmacology and Molecular Sciences Johns Hopkins University School of Medicine Baltimore Maryland USA; ^3^ Johns Hopkins Drug Discovery and Department of Neurology Johns Hopkins University School of Medicine Baltimore Maryland USA

## Abstract

**Rationale:**

Glutamate carboxypeptidase II (GCPII) catalyzes the hydrolysis of *N*‐acetylaspartylglutamate (NAAG) to yield glutamate (Glu) and *N*‐acetylaspartate (NAA). Inhibition of GCPII has been shown to remediate the neurotoxicity of excess Glu in a variety of cell and animal disease models. A robust high‐throughput liquid chromatography–tandem mass spectrometry (LC/MS/MS) method was needed to quantify GCPII enzymatic activity in a biochemical high‐throughput screening assay.

**Methods:**

A dual‐stream LC/MS/MS method was developed. Two parallel eluent streams ran identical HILIC gradient methods on BEH‐Amide (2 × 30 mm) columns. Each LC channel was run independently, and the cycle time was 2 min per channel. Overall throughput was 1 min per sample for the dual‐channel integrated system. Multiply injected acquisition files were split during data review, and batch metadata were automatically paired with raw data during the review process.

**Results:**

Two LC sorbents, BEH‐Amide and Penta‐HILIC, were tested to separate the NAAG cleavage product Glu from isobaric interference and ion suppressants in the bioassay matrix. Early elution of NAAG and NAA on BEH‐Amide allowed interfering species to be diverted to waste. The limit of quantification was 0.1 pmol for Glu. The *Z*‐factor of this assay averaged 0.85. Over 36 000 compounds were screened using this method.

**Conclusions:**

A fast gradient dual‐stream LC/MS/MS method for Glu quantification in GCPII biochemical screening assay samples was developed and validated. HILIC separation chemistry offers robust performance and unique selectivity for targeted positive mode quantification of Glu, NAA, and NAAG.

## 1 INTRODUCTION

**FIGURE 1:**
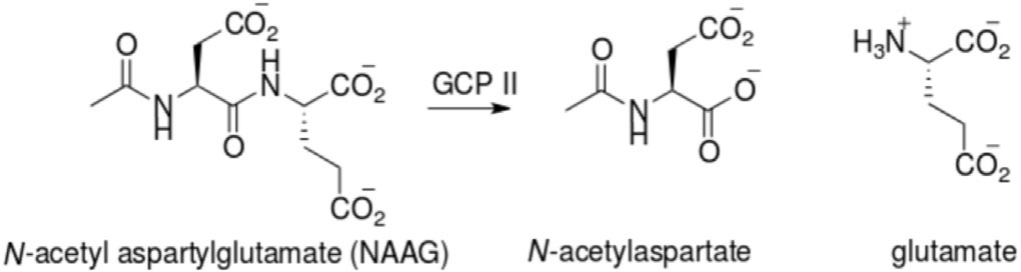
GCPII reactants at physiological pH.

*N*‐acetylaspartylglutamate (NAAG) is the most abundant dipeptide in the brain, providing strict metabolic control of glutamate (Glu) through activation of the type 3 metabotropic glutamate receptor.^1^ Glutamate carboxypeptidase II (GCPII) catalyzes the hydrolysis of NAAG to yield Glu and *N*‐acetylaspartate (NAA) (Figure 1). Due to its role in modulating levels of NAAG and Glu, GCPII is a promising therapeutic target for many diseases and conditions, including stroke, traumatic brain injury, epilepsy, age‐related neurodegenerative diseases, schizophrenia, and pain.^2^ Inhibition of GCPII has been shown to remediate the neurotoxicity of excess Glu in a variety of cell and animal disease models.^3^, ^4^, ^5^ Diverse GCPII inhibitors have been available from as far back as 1988, but they universally fail to pass the blood–brain barrier. Accordingly, they are not therapeutically viable for CNS diseases.^3^, ^6^ The National Center for Advancing Translational Sciences (NCATS) partnered with Johns Hopkins University (JHU) to identify small‐molecule GCPII inhibitors likely to have improved CNS penetrance as part of the Helping to End Addiction Long‐term (HEAL) initiative. For this research, a robust, high‐throughput, and economically efficient GCPII assay was required for screening. Although several GCPII activity assays have been developed, pre‐existing assays had drawbacks that made them unsuitable for high‐throughput screening. The well‐established radiometric assay for GCPII was sufficiently precise but not operationally suitable for high‐throughput screening and would risk exposing high‐throughput machinery to radioactive compounds.^5^ The commercially available Amplex Red glutamic acid kit (Invitrogen Corp., CA, USA) relied on indirect assessment of GCPII activity by utilizing glutamate oxidase and horseradish peroxidase catalyzed fluorescence. Screening with such an assay would risk generating false hits targeting glutamate oxidase, targeting horseradish peroxidase, and/or scavenging hydrogen peroxide. Accordingly, we developed a high‐throughput liquid chromatography–tandem mass spectrometry (LC/MS/MS) method to directly measure Glu resulting from *in vitro* GCPII cleavage of NAAG.

Despite the abundance and functional importance of Glu, NAAG and NAA, existing targeted LC/MS/MS methods for their bioanalysis are quite varied and lack sufficient throughput for high‐throughput screening.^6^, ^7^, ^8^ Glu is a small and highly hydrophilic molecule that does not retain well in reverse‐phase chromatography (RP‐HPLC). Nevertheless, several RP‐HPLC methods for Glu analysis have been developed, most of which utilize derivatization and high salt concentrations to improve retention on C_18_ stationary phases.^9^, ^10^ In contrast to RP‐HPLC, HILIC (hydrophilic interaction liquid chromatography) stationary phases are polar, typically prepared as bare silica or silica gels modified with many polar functional groups.^11^ Retention mechanisms in HILIC are multi‐modal, a combination of hydrophilic, ion‐exchange and reverse‐phase interactions. Stationary phase chemistry can be tailored to provide unique selectivity for separation of small polar structurally related chemical species.^12^, ^13^

Herein, we describe the development of a dual‐channel high‐throughput LC method that was used for quantification of Glu in a biochemical screening assay. The method was used to screen small‐molecule libraries totaling ~36 000 compounds for inhibitors of GCPII at a throughput of one sample per minute with a mean *Z*‐factor of 0.85.^14^ We report on method development, including the profiling of Penta‐HILIC and BEH‐Amide sorbent chemistries for separation of Glu, NAA, and NAAG.

## 2 MATERIALS AND METHODS

### 2.1 Chemicals and reagents

LC/MS‐grade acetonitrile, water, and formic acid were purchased from ThermoFisher (Waltham, MA). D_3_‐glutamate,^13^C_5_^15^N‐acetylaspartylglutamate, ^13^C_5_^15^N‐glutamate, *N*‐acetylaspartic acid, NAAG, and Glu were purchased from Sigma‐Aldrich (St Louis, MO). GCPII was purchased from Sino Biological (Wayne, PA). Clear, polypropylene, flat‐bottom 384‐well plates were purchased from Greiner Bio‐One (Monroe, NC).

### 2.2 Biochemical assay

The biochemical assay utilized an enzymatic buffer, a compound plate, and a quenching solution. The enzymatic buffer consisted of 5 μL of GCPII enzyme prepared in Tris–HCl (pH 7.4) with 1mM ZnCl_2_. The compound plate consisted of test compounds acoustically dispensed from 10mM DMSO stocks to columns 3–16 of a polypropylene 384‐well plate containing 5 μL of ^13^C_5_^15^N‐acetylaspartylglutamate also prepared in Tris–HCl (pH 7.4) with 1mM ZnCl_2_. Into columns 1 and 2 was dispensed an equal volume of DMSO. A Beckman‐Coulter BioRaptr nanovolume dispenser was utilized to transfer the enzymatic buffer to columns 2–16 of the compound plate, and the reaction was allowed to proceed for 30 min while shaking gently. After 30 min, 50 μL of 0.2 μM D_3_‐glutamate suspended in LC/MS‐grade acetonitrile with 0.1% formic acid was added to the sample plate to quench the reaction and incorporate the internal standard. The resulting sample is described in Table 1.

**TABLE 1:** GCPII/NAAG assay protocol components and volumes (per well in a 384‐well plate).

Reagent	Volume (μL)
40mM Tris–HCl pH 7.4, 1mM ZnCl_2_, 1 nM GCPII	5
NAAG 500 nM (prepared in water)	5
Reaction volume	10
Acetonitrile quench	70
0.1% formic acid	
0.2 μM ^3^D‐glutamate	
Total volume per well for LC/MS	80

### 2.3 HPLC sample delivery system

**FIGURE 2:**
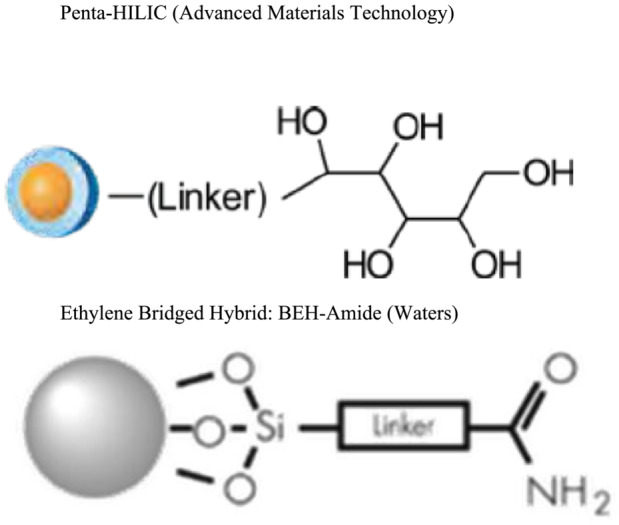
BEH‐Amide and Penta‐HILIC sorbent chemistries assessed in method development. [Color figure can be viewed at wileyonlinelibrary.com]

**FIGURE 3:**
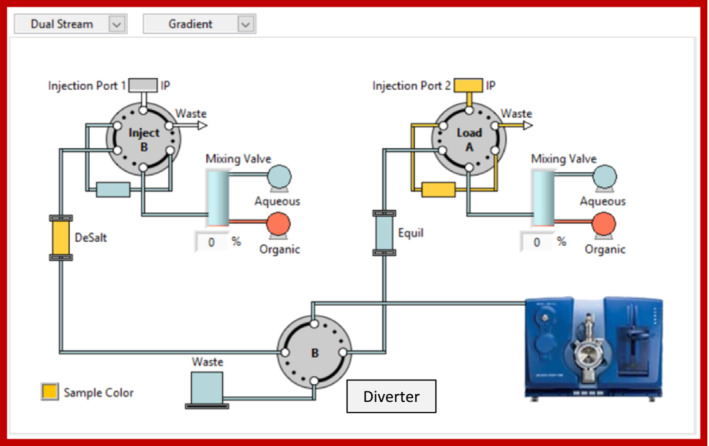
Dual‐stream plumbing diagram as shown within LeadScape™ software. The diverter valve switches the sampled stream at the mid‐point of the gradient cycle. [Color figure can be viewed at wileyonlinelibrary.com]

The sample delivery system consisted of two model 1290 ultrahigh‐performance binary liquid gradient pumps (Agilent, Santa Clara, CA) coupled with a lead sampler 1 (LS‐1) autosampler (Sound Analytics, Niantic, CT). Two column chemistries were tested (Figure 2): BEH‐Amide, 3.5 μm, 2.1 × 30 mm (Waters, Milford, MA) and HALO Penta‐HILIC, 2.7 μm, 2.1 × 30 mm (Mac‐Mod Analytical, Chadds Ford, PA). The optimized LC gradient conditions are listed in Table 2. Mobile phase A consisted of 0.05% formic acid in water; mobile phase B was acetonitrile. Cycle time was 2 min per LC channel. The LS‐1 was configured such that injection port 1 was designated channel 1 and injection port 2 was designated channel 2. A two‐position valve (Rheodyne/IDEX, Rohnert Park, CA) was used to schedule sampling between channels as diagramed in Figure 3. The overall cycle time in dual‐channel sampling mode was 1 min per sample.

**TABLE 2:** Liquid chromatography and gradient conditions.

Columns	Waters Xbridge BEH‐Amide, 2 × 30 mm, 3.5 μm
	Halo Penta‐HILIC, 2 × 30 mm, 2.7 μm
Flow rate:	0.7 mL/min
Mobile phase A:	0.05% formic acid (pH 2.7)
Mobile phase B:	Acetonitrile
Time (min)	B (%)
0	84
0.55	84
0.6	45
0.65	45
0.7	84
2	84

### 2.4 MS/MS conditions and settings

A Sciex 6500+ QTrap mass spectrometer (Sciex, Toronto, CN) was used for all sample analysis. Nitrogen was used as collision and curtain gas. The source parameters were ion spray voltage: 5200 V; Turbo‐V source gas 1: 50 psi; source gas 2: 50 psi; and probe temperature: 400°C. The Glu, *N*‐acetylaspartic acid, and NAAG transitions and collision energies were acquired using the LeadScape automated tuning function, optimizing for maximum sensitivity of the MS/MS transitions used (Table 3).

**TABLE 3:** MS/MS settings.

Analyte	M + 1	MS/MS	DP	CE
^13^C^15^N‐Glu	154	89	30	21
^3^D‐glutamate	151	87	30	20
NAA	176	134	60	13
NAAG	305	148	80	14
l‐Asp	134	74	55	19

### 2.5 Software setup and data processing for dual‐stream bioanalysis

LeadScape™ software (Sound Analytics, Niantic, CT) was used for system control and data processing. The “Import Text File with Positions” utility within LeadScape was used to set up dual‐stream analysis. Injection sequences were organized by row. Stream 1/row 1, and stream 2/row 2, alternated sampling such that each stream sampled eight rows per 384‐well plate (stream 1 sampled rows: A, C, E, G, I, K, M, O; and stream 2 sampled rows: B, D, F, H, J, L, N, P). Sample names and positions (plate location and well ID) were imported into LeadScape's Analyze module to assign the LS‐1's sampling sequence. During analysis a batch data file having extension *.bdf is created. The bdf file contains metadata (sample name and position). At the conclusion of a run the bdf file is imported to start the data review process. Batch setup, data review, and export are described in Figure S2. The ratio of the ^13^C_5_^15^N‐glutamate peak to the D_3_‐glutamate peak was used for normalized quantitation of the enzymatic turnover. Column 1, consisting of the reaction mix without the GCPII enzyme, was used as a negative control. Column 2, consisting of the reaction mix without any test compound, was used as the positive control. The inhibitory activity of test compounds was indicated by reactions with reduced enzymatic turnover as compared to the positive control in the same row.

## 3 RESULTS AND DISCUSSION

### 3.1 Method development

Throughput and robust performance were primary requirements in developing an LC/MS/MS method to support the GCPII screening campaign. Throughput is an important criterion in selecting a screening strategy that is both relevant to the target biology and allows testing of an adequately large number of compounds to identify hits that can act as starting points for medicinal chemistry. Predictive and chemoinformatic approaches can be used to assemble small‐molecule libraries of sufficient size suitable for profiling chemical space in productive timeframes.^15^, ^16^, ^17^, ^18^ Table 4 lists LC/MS analysis time and associated throughput. Acoustic‐ejection mass spectrometry (AEMS) is an emerging technology featuring very high‐throughput, label‐free MS/MS detection and low signal‐to‐background response.^19^, ^20^ AEMS should reduce costs and improve the quality of large‐scale high‐throughput screening campaigns.^21^ However, like trap and elute methods, AEMS is not able to separate analytes; it relies on sample dilution to reduce signal interference and suppression. We focused on more traditional LC/MS‐based screening methods with the expectation to screen libraries containing 3000 to 10 000 discrete chemical entities in singleton format.

**TABLE 4:** Per sample throughput versus number of samples.

Sample introduction technology (min/sample)	Analysis time (h)/number of samples		
Number of samples >	1000	4000	10 000
Traditional LC (2 min)	33	133	333.3
LS‐1 dual‐stream HILIC (1 min)^22^, ^23^	17	67	166.7
Ballistic gradient (0.5 min)	8	33	83.3
Trap‐and‐elute^22^, ^23^	3	13	33.3
Acoustic Droplet Ejection^19^, ^20^, ^21^	0.3	0.9	3.3

**FIGURE 4:**
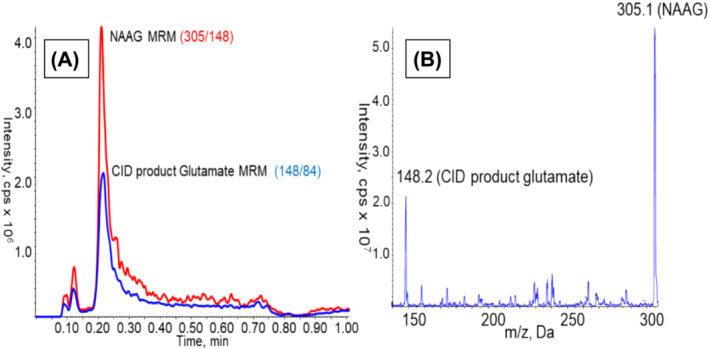
(A) Co‐elution of NAAG (305/148 *m*/*z*) and its collisionally induced fragment ion Glu (148/84 *m*/*z*) at 0.25 min retention time using the HILIC method described in Table 2 on BEH‐Amide. The NAAG sample was prepared as a standard in bioassay matrix (Table 1) absent of GCPII with the quench mix used as diluent. A discrete Glu peak was not present at 0.7 min demonstrating that Glu is formed post‐column and is not present at significant concentrations as a NAAG contaminant. (B) Full‐scan spectra of 1 μM NAAG standard infused at a flow rate of 0.7 mL/min in 84% acetonitrile illustrating the up‐front collision‐induced dissociation of NAAG to Glu. [Color figure can be viewed at wileyonlinelibrary.com]

In some cases, trap‐and‐elute methods work well for biochemical assay screening with cycle time ranging from 10 to 20 s per sample. This approach relies on an on‐line desalting step that diverts early eluting and non‐volatile substances to waste, thereby reducing signal suppression and helping to keep the ionization source clean. Biochemical assay matrices are generally composed of an enzyme, biological buffer, and salts; these components are not retained by the HILIC sorbent and elute in the column void volume. The desalting step is critical in trap‐and‐elute since retained analytes and matrix components elute in a single band and there is little or no separation. The lack of separation can result in signal suppression and the potential for isobaric interference is thus much greater. In the case of the NAAG–GCPII reaction, collision‐induced dissociation of NAAG in the source region releasing free Glu precluded the use of trap‐and‐elute methods due to *m*/*z* overlap with enzymatically generated Glu (Figure 4). Further optimization of the declustering potential, source temperature, and electrospray probe voltage was insufficient to eliminate up‐front collision‐induced dissociation of the NAAG substrate. Separation of Glu and NAAG was thus required to successfully use an ESI‐LC/MS/MS method.

### 3.2 Development of a HILIC‐based separation method

HILIC has established itself as the separation mode of choice for highly hydrophilic and amphiphilic compounds that are too polar to be retained in RP‐LC but have charge density sufficient for electrostatic and/or ion‐exchange interactions to drive separations chemistry. We assessed Penta‐HILIC and BEH‐Amide sorbents to determine the most suitable separation chemistry for high‐throughput quantification of Glu. The columns tested had equivalent dimensions, 2.1 × 30 mm, with 2.7 μm or 3.5 μm particle sizes for Penta‐HILIC and BEH‐Amide, respectively. Aqueous, with 0.05% formic acid modifier, and acetonitrile mobile phases were used (pH 2.7) along with fast linear gradients, 1–2 min cycle times, to assess separation efficiency and peak shape.

Signal suppression could potentially be reduced by volumetric dilution during the assay quench step. Glu's ionization efficiency in positive mode is relatively low, however, and further dilution would likely impact the limit of quantification. Therefore, method development focused on establishing a clean elution window for Glu to maximize signal response and avoid co‐elution with NAAG and NAA.

Initial testing with Penta‐HILIC sorbent used the methodology described by Sangaraju et al.^24^ The Penta‐HILIC stationary phase consists of a silane‐linked sorbitol moiety containing five aliphatic hydroxyl groups. The sorbent is designed to facilitate the formation of an evenly distributed and consistent water layer in association with the silica bonded phase. Hydrogen bonding and dipole interactions between sorbent hydroxyl groups and aqueous mobile phase serve to maintain the water layer thereby enhancing solute partitioning interactions between the non‐polar mobile phase and an aqueous stationary phase. The partitioning mode was designed to resemble a reverse‐phase separation wherein solutes separate based on their amphiphilic nature.^25^

Although the Penta‐HILIC phase provided baseline separation of Glu, NAA and NAAG and reasonable throughput (45 s per sample), performance was limited by signal suppression and high background during analysis of bioassay samples. Glu elutes prior to NAA and NAAG on Penta‐HILIC. This elution order is somewhat surprising since Glu is the more hydrophilic substrate. Based on its size and distribution coefficient at pH 2.7, we might expect it to be the more strongly retained substrate in a predominantly HILIC‐based retention mechanism (Table 5 lists physicochemical properties for the substrates). The elution order is reversed on the BEH‐Amide phase with NAA and NAAG eluting prior to Glu. Glu's peak shape and retention factor were nearly identical on both column chemistries when using the same mobile phase and gradient conditions. The elution of NAA and NAAG prior to Glu on BEH‐Amide allowed adjustment of gradient conditions such that the initial percent aqueous was able to flush the majority of NAA and NAAG to waste while retaining Glu. While this approach removed signal suppression and reduced background, the cycle time was 2 min per sample, too slow to support a large‐scale screening effort. To reduce cycle time a dual‐stream LC method was developed.

**TABLE 5:** Calculated physicochemical properties of analytes.

Analyte	p*K*_a1_ COOH	p*K*_a_ NH_2_	p*K*_a2_ COOH	p*K*_a3_ COOH	LogD (pH 2.7)	LogP	HBD	HBA	TPSA (Å)	Rotatable bonds
Glu	2.1	9.5	4.07	—	−6.7	−3.7	3	5	100.6	4
NAA	3.4	—	5.3	—	−1.5	−1.4	3	5	103.7	4
NAAG	3.7	—	4.3	3.1	−2.4	−2.3	5	8	170.1	9
l‐Asp	2	9.4	3.9	—	−6.9	−3.9	3	5	100.6	3

### 3.3 Dual‐stream LC/MS/MS methodology

**FIGURE 5:**
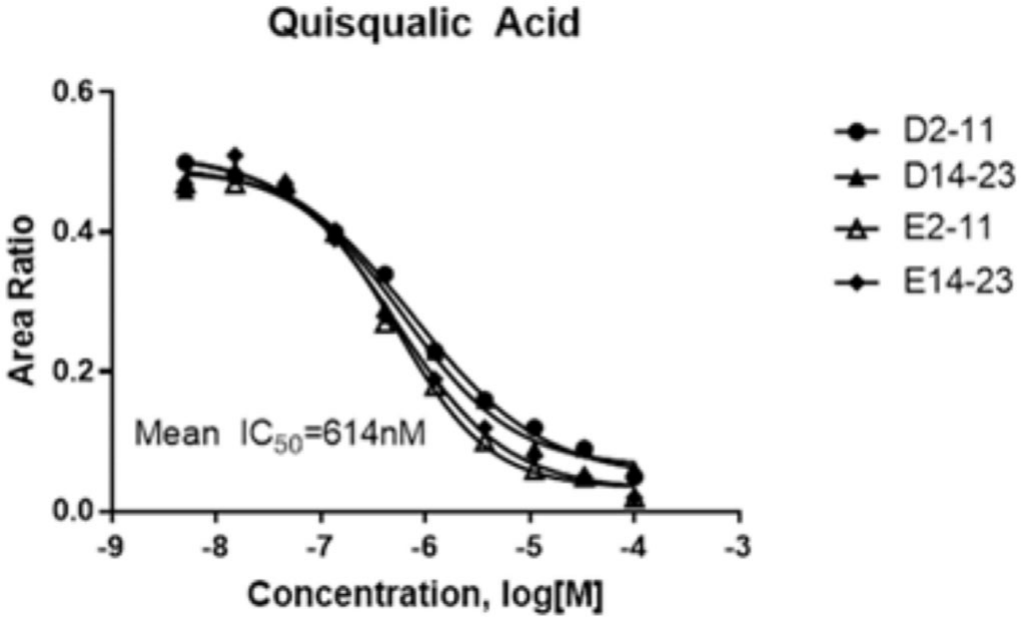
IC_50_ curves of the known GCPII inhibitor quisqualic acid taken to assess intra‐assay variation. Row D was injected through stream one of the dual‐stream method while row E was injected through stream two. The experimental IC_50_ is in good agreement with previously reported values^27^, ^28^ and the two streams provide comparable and consistent results.

To increase throughput without sacrificing chromatographic resolution of Glu and NAAG, the single‐channel method was adapted for dual‐stream analysis. Two Agilent 1290 infinity UHPLC pumps were used to run two discrete LC channels on the LS‐1 autosampler (Figure S1 depicts LC/MS/MS dual‐stream workstation). The “Duality” dual‐stream scheduling utility within LeadScape™ system software enabled routine screening of three complete 384‐well plates per day at a throughput of 1 min per sample. The cycle time per channel was 2 min, which reduced to 1 min when eluent streams were combined. Chromatographic parallelization is an effective means of increasing throughput without necessitating sacrifices in sensitivity or quantitative reproducibility associated with fast LC/MS analysis.^26^ The Duality mode within LeadScape™ is a staggered parallel analysis method; subsequent injections are scheduled at the midpoint of the injection cycle.^22^ This approach works well for fast linear gradient cycle times of 2 min or less. Cycle time per channel may need to be increased if there are very late or very early eluting peaks to account for. Each channel comprises a discrete LC system that includes binary gradient pump, injection port, and column. A two‐position UHPLC valve schedules MS/MS sampling from each eluent stream. In singleton screening applications the batch file was formatted to sample from 384‐well plates by row. For example, plate rows A, C, and E were sampled through LC channel 1 and rows B, D, and F were sampled through LC channel 2. There were a total of 50 injections per acquisition (.wiff) file (24 injections for each row plus two BLK‐ISTD samples). Although samples from two rows were collected in each acquisition file, each row of samples was reviewed separately and interactively as a group. LeadScape software automatically splits the acquisition file into A and B channels during data review. A “bdf” (batch data file, extension .bdf), generated at batch submission, contains metadata for each sample and includes information on the dual‐stream sampling sequence and how acquisition files are split for data review. This feature was used to review peak response data in context of the LC channel it was run on. As illustrated in Figure 5, we see performance consistency across peaks associated with a specific LC pump and column. For example, a combination of peak shape, retention time, and ISTD response variability (relative standard deviation) was used to assess performance across singleton screening runs. Hit follow‐up studies were organized to evaluate an IC_50_ curve as reflected in peak response for a single analyte per channel (Figure S2 describes the dual‐stream batch setup through data review workflow).

### 3.4 Discussion

We focused on HILIC retention chemistry in developing an analytical method to measure Glu at sufficient throughput to support small‐molecule GCPII hit identification screening. Initial method development was hampered by signal interference co‐eluting with Glu that reduced the dynamic range of the biochemical assay. Initial “trap and elute” methods had poor *Z*‐factor scores, *Z* = 0.5 at best. It was subsequently learned that the interference was due to a combination of in‐source dissociation of the NAAG dipeptide releasing free Glu, and ESI signal suppression due to co‐elution of Glu, NAAG, and NAA metabolites. This finding precluded the use of trap and elute LC methods where there is little or no separation of retained substrates.^29^, ^30^ Co‐elution of NAAG, its collision‐induced fragment Glu, and enzymatically generated Glu meant that chromatographic separation of the analytes was required for accurate quantification. We pursued development of a fast gradient LC method to separate NAAG, NAA, and Glu. An understanding of retention behavior of each metabolite was important to avoid co‐elution and find a clean elution window for Glu. Two HILIC column chemistries were tested, BEH‐Amide and Penta‐HILIC, in column format, 2 × 30mm, 2.7 and 3.5 μm particle size for Penta‐HILIC and BEH‐Amide, respectively.

**FIGURE 6:**
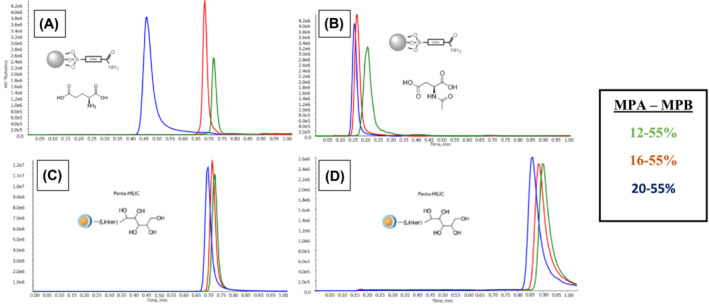
Elution profile of NAA and Glu on BEH‐Amide and Penta‐HILIC sorbents. Three step gradient profiles were tested: 12% (green trace), 16% (red trace), and 20% (blue trace) aqueous. The gradient used (Table 1) features a 0.55 min hold at low percent aqueous prior to stepping up the 55% aqueous for elution. Glu elutes much earlier and its peak shape is significantly broadened at 20% aqueous on the BEH‐Amide phase (A). The more lipophilic NAA's peak shape is broadened at low percent aqueous, higher organic content (B). These observations suggest that solute physicochemical and structural properties predict Glu and NAA retention on the BEH‐Amide phase. The stronger retention of relatively polar, but less hydrophilic, NAA compared to Glu suggests that two discrete retention mechanisms are active on Penta‐HILIC stationary phase (C, D) under these conditions. Glu's hydrophilicity allows it to interact directly with the stationary phase being retained by predominantly charge‐based polar/dipole interaction. In contrast, we hypothesize that NAA is predominantly retained by hydrogen bonding interactions with the water‐enriched sorbitol layer on Penta‐HILIC. [Color figure can be viewed at wileyonlinelibrary.com]

Retention in HILIC is typically proportional to analyte hydrophilicity.^31^ Glu and Asp are strongly hydrophilic (predicted LogD of −6.7 and −6.9 at pH 2.7 for Glu and Asp, respectively), they are well retained, and their elution order is maintained for both BEH‐Amide and Penta‐HILIC sorbent chemistries, the more hydrophilic Asp eluting slightly later than Glu. It is noteworthy that the more lipophilic substrates NAA and NAAG elute after Glu on the Penta‐HILIC sorbent. The four metabolites tested have similar structural and physicochemical properties (see Table 5). The *N*‐acetyl group exerts significant influence on Asp, NAAG, and NAA retention for Penta‐HILIC in comparison to BEH‐Amide sorbent. To better understand retention chemistry, a study was run comparing eluent strength across the two phases. The gradient A/B schedule and cycle times were kept constant while the initial percent aqueous mobile phase was increased. Figure 6 depicts separation chemistry for Glu and NAA at 12%, 16%, and 20% initial aqueous mobile phase. Retention time and peak shape for Glu and NAA were affected to a greater degree on the BEH‐Amide phase compared to Penta‐HILIC. Glu's retention factor was halved, *R*_f_ = 6.8 at 16% aqueous to 3.4 at 20%, and peak shape broadened significantly (Figure 6A) suggesting an ion exchange‐like Glu–BEH‐Amide interaction. In contrast, NAA peak shape was sharpened at 20% aqueous and broadened significantly at 12% (Figure 6B), the higher percent organic mobile phase component inducing band broadening of the more lipophilic NAA substrate. These results suggest that solute interactions are predominantly charge‐based for these analytes on BEH‐Amide and lipophilicity is a good predictor of retention strength. In comparison, retention time and peak shape do not change that much on the Penta‐HILIC sorbent as organic content is increased (Figures 6C and 6D). The Penta‐HILIC phase appears to add a second retention mechanism that strongly retains less polar analytes like NAA, whose structural features favor more of an aqueous partitioning and hydrogen bonding retention mechanism as proposed by Persson et al.^25^ The *N*‐acetyl group is sufficient to dominant retention on Penta‐HILIC while unmodified Asp elutes much earlier and is predominantly retained by charge interaction.

In addition, we tested the use of mobile phase flow rate to shorten column re‐equilibration time and increase throughput. Increasing equilibration volume has been demonstrated to reduce runtime for HILIC‐based LC methods.^32^ We found flow rate to be an important component in maintaining peak shape and consistency throughout the 6 to 7 h per 400 sample runtimes (i.e., 384‐well plate plus 16 injections of QC samples at 1 min per sample throughput). Attempts to reduce re‐equilibration time by increasing flow rate to 1.0 mL/min did not sufficiently increase throughput to justify the deleterious effect on peak shape when compared to maintaining flow rate at 0.7 mL/min nor the increased complexity of processing multiply injected files with irregular run times. A 2 min cycle time with 2.1 × 30 mm column and 0.7 mL/min flow rate resulted in about 10–12 column re‐equilibration volumes. Although consistent “partial” re‐equilibration has been shown to be effective in shortening cycle time when using HILIC gradient methods, we were not able to use this approach.^33^ Shortening the re‐equilibration time led to suppression of Glu response. Despite diverting NAAG and NAA to waste, even minor incremental reduction in equilibration volume resulted in signal suppression. This finding suggests that the electronically active HILIC sorbent phase retains salt and or related ionic components strongly in comparison to reverse‐phase sorbents. We did not specifically test the addition of mobile phase modifiers that may decrease equilibration time by disrupting charge‐based matrix interactions.

The methodology described was used for primary and hit follow‐up screening of over 36 000 small molecular entities. The bridged ethylene hybrid stationary phase adds structural strength and stability to the BEH‐Amide sorbent.^34^, ^35^ BEH‐Amide separation and sorbent phase chemistries proved very robust, typically achieving over 2500 injections per column in dual‐column configuration. This allowed completion of large screening sets without needing to change columns.

The advantages of a two‐column dual‐stream LC/MS/MS system and single‐sprayer mass spectrometer have been described,^36^ its primary strength being simplicity. While four‐channel LC systems can achieve greater absolute throughput, valve scheduling, LC plumbing, and system maintenance can limit flexibility and routine use.^37^, ^38^ The application described does not have scheduling options; it was designed for parallel analysis of identical LC methods each having the same cycle time.^23^, ^39^ Addition of the second LC stream cuts per channel cycle time by half, doubling throughput. There are no other options, each eluent stream is a discrete run. This operational construct is carried through the 384‐well plate sampling sequence and data acquisition.

The dual‐stream LC/MS/MS method increased throughput and proved robust throughout the screening campaign. The sample delivery hardware and software automation required to organize 384‐well plate‐based analysis and complete data processing was easily adapted to internal endpoint calculation and database tracking processes. The method returned high‐quality data with *Z*‐factor values that averaged better than 0.8 across several months of screening.

## AUTHOR CONTRIBUTIONS

*Conceptualization*: Barbara S. Slusher, John S. Janiszewski, Rick Schneider, Anu Dalal, Yixuan Qiu, and Stephen C. Kales. *Methodology*: John S. Janiszewski, Yixuan Qiu, Nate Hoxie, Rick Schneider, Stephen C. Kales, Xin Hu, and Huijun Wei. *Software*: N/A. *Validation*: Robyn Wiseman, BSB, Takashi Tsukamoto. *Formal analysis*: Xin Hu. *Investigation*: Nate Hoxie, John S. Janiszewski, Yixuan Qiu, and Stephen C. Kales. *Resources*: Matthew D. Hall, BSB, Xin Hu, and Stephanie L. Ford‐Scheimer. *Data curation*: Nate Hoxie, John S. Janiszewski, and Xin Hu. *Writing—original draft*: Nate Hoxie and John S. Janiszewski. *Writing—review and editing*: Nate Hoxie, John S. Janiszewski, Matthew D. Hall, BSB, Takashi Tsukamoto, Robyn Wiseman, Xin Hu, and Stephen C. Kales. *Visualization*: Nate Hoxie and John S. Janiszewski. *Supervision*: John S. Janiszewski, Matthew D. Hall, BSB, and Stephanie L. Ford‐Scheimer. *Project Administration*: Stephanie L. Ford‐Scheimer and Matthew D. Hall. *Funding acquisition*: Matthew D. Hall, BSB, and Stephanie L. Ford‐Scheimer.

4

### PEER REVIEW

The peer review history for this article is available at https://www.webofscience.com/api/gateway/wos/peer-review/10.1002/rcm.9772.

## Supporting information


**Figure S1.** Dual‐Stream plumbing; Injection ports 1 and 2 (as labeled; upper left and right in pic) comprise two identical, independent LC systems. Each system runs the same gradient method and cycle time. Cycle time is 2 min for the GCPII HILIC BEH‐Amide method. Valve three (center) coordinates sampling from each stream. At the outset both systems are equilibrated and running initial conditions. Channel 1’s cycle starts when a sample is injected. Valve 3 switches at the midpoint of the cycle time, to direct Channel 2 eluent to the mass spectrometer. This timing offsets injections by 60 sec, resulting in an overall throughput of about 1 min per sample. Either system can be run independently at a throughput of 2 min per sample. All LC tubing lengths are identical. Each sample loop is 8 μL volume (labeled SL1 and SL2 above). Likewise, transfer tubing between columns (Labeled 1 and 2) have identical lengths and internal diameter (ID = 0.005″).
**Figure S2a**. The “Text Import File” is a user organized list of information needed for dual‐stream LC/MS/MS analysis on the LS‐1 autosampler, information such as; sampling sequence, compounds and project associated meta data is included. The Annotations below table are in context of GCPII method for a 26‐sample batch (two rows of 96‐well plate plus two “BLK + ISTD” samples,. The Header Row (labeled 1 at top row of Fig.) syncs with a method specific import format set up in LeadScape. The file is loaded into the Batch Queue to start the run. During runtime, this information is accumulated and stored in a “*.bdf” file (batch data file) along with real time information needed to process the run. At the conclusion of a run, the *.bdf file is read into LeadScape for data processing.
**Figure S2b**. The ‘Preview Batch’ window lists the specific sequence of injections used by the autosampler. Rows 1, 3, 5 etc. are run through Channel‐1 (LC system 1) Rows 2, 4, 6 etc. are run through channel 2 (LC system 2). There are 26 injections in the acquisition file depicted above. During data review the batch information is used to split the acquisition window in half such that 13 peaks are viewed together as a coherent group all acquired on the same LC system/channel A 384‐well sampling batch has an identical by row sampling format, except the numbers of samples acquired per file increases from 26 to 50 as 2x12‐well rows, expand to 2x24‐well rows for 384 W plate (**see slides S2c, d**).
**Figure S2c**. Select Batch to Review: User selects a batch data file (extension *.bdf) for review (label **1**). The bdf file splits the acquisition window by channel depending on information and format of the text file submitted to the queue at run time (**Figure S2a**). The bdf file has all the information needed to process the data and export results in csv format.
**FigureS2d**. LeadScape Dual‐Stream review. The review window shows Stream 1, Row E's chromatographic trace and data table for the internal standard (D3‐Glu) transition, 151/87 *m/z* (Label 2). There are 25 peaks corresponding to Row E samples (1–22) plus separate BLK + ISTD injection. **Label 1:** Once the data set is loaded LCMS data is reviewed by stream, **Label 2**: Select Internal standard MS/MS transition (151/87 *m/z*), **3:** Row E, 25 total injections (24 sample wells and BLK + ISTD sample)
**Figure S2e**. LeadScape Dual‐Stream review: Stream 2’s chromatographic trace and data table for analyte (13C5‐Glu) transition 153/88 *m/z*. **Label 1:** Reviewing set1‐Stream 2, **Label 2**: Select 13C5‐Glu MS/MS transition, **Label 3:** Row D, 25 total injections (24 sample wells and BLK + ISTD sample)
**Figure S5:** 11‐point IC50s collected for the known GCPII inhibitor quisqualic acid, taken to assess stream‐to‐stream reproducibility. The resulting IC_50_ (1.59 +/− 0.17 μM, 95% CI) agrees with previously reported values
