# Supplementary material for: Development of a high‐throughput dual‐stream liquid chromatography–tandem mass spectrometry method to screen for inhibitors of glutamate carboxypeptidase II
Source: Rapid Commun Mass Spectrom. 2024 Jun 12;39(Suppl 1):e9772. doi: 10.1002/rcm.9772 (PMC11737842; doi:10.1002/rcm.9772)
Supplement: Supplementary file 1 — Figure S1. Dual‐Stream plumbing; Injection ports 1 and 2 (as labeled; upper left and right in pic) comprise two identical, independent LC systems. Each system runs the same gradient method and cycle time. Cycle time is 2 min for the GCPII HILIC BEH‐Amide method. Valve three (center) coordinates sampling from each stream. At the outset both systems are equilibrated and running initial conditions. Channel 1’s cycle starts when a sample is injected. Valve 3 switches at the midpoint of the cycle time, to direct Channel 2 eluent to the mass spectrometer. This timing offsets injections by 60 sec, resulting in an overall throughput of about 1 min per sample. Either system can be run independently at a throughput of 2 min per sample. All LC tubing lengths are identical. Each sample loop is 8 μL volume (labeled SL1 and SL2 above). Likewise, transfer tubing between columns (Labeled 1 and 2) have identical lengths and internal diameter (ID = 0.005″). Figure S2a. The “Text Import File” is a user organized list of information needed for dual‐stream LC/MS/MS analysis on the LS‐1 autosampler, information such as; sampling sequence, compounds and project associated meta data is included. The Annotations below table are in context of GCPII method for a 26‐sample batch (two rows of 96‐well plate plus two “BLK + ISTD” samples,. The Header Row (labeled 1 at top row of Fig.) syncs with a method specific import format set up in LeadScape. The file is loaded into the Batch Queue to start the run. During runtime, this information is accumulated and stored in a “*.bdf” file (batch data file) along with real time information needed to process the run. At the conclusion of a run, the *.bdf file is read into LeadScape for data processing. Figure S2b. The ‘Preview Batch’ window lists the specific sequence of injections used by the autosampler. Rows 1, 3, 5 etc. are run through Channel‐1 (LC system 1) Rows 2, 4, 6 etc. are run through channel 2 (LC system 2). There are 26 injections in the acqui [file RCM-39-e9772-s001.pptx]

## Slide 1
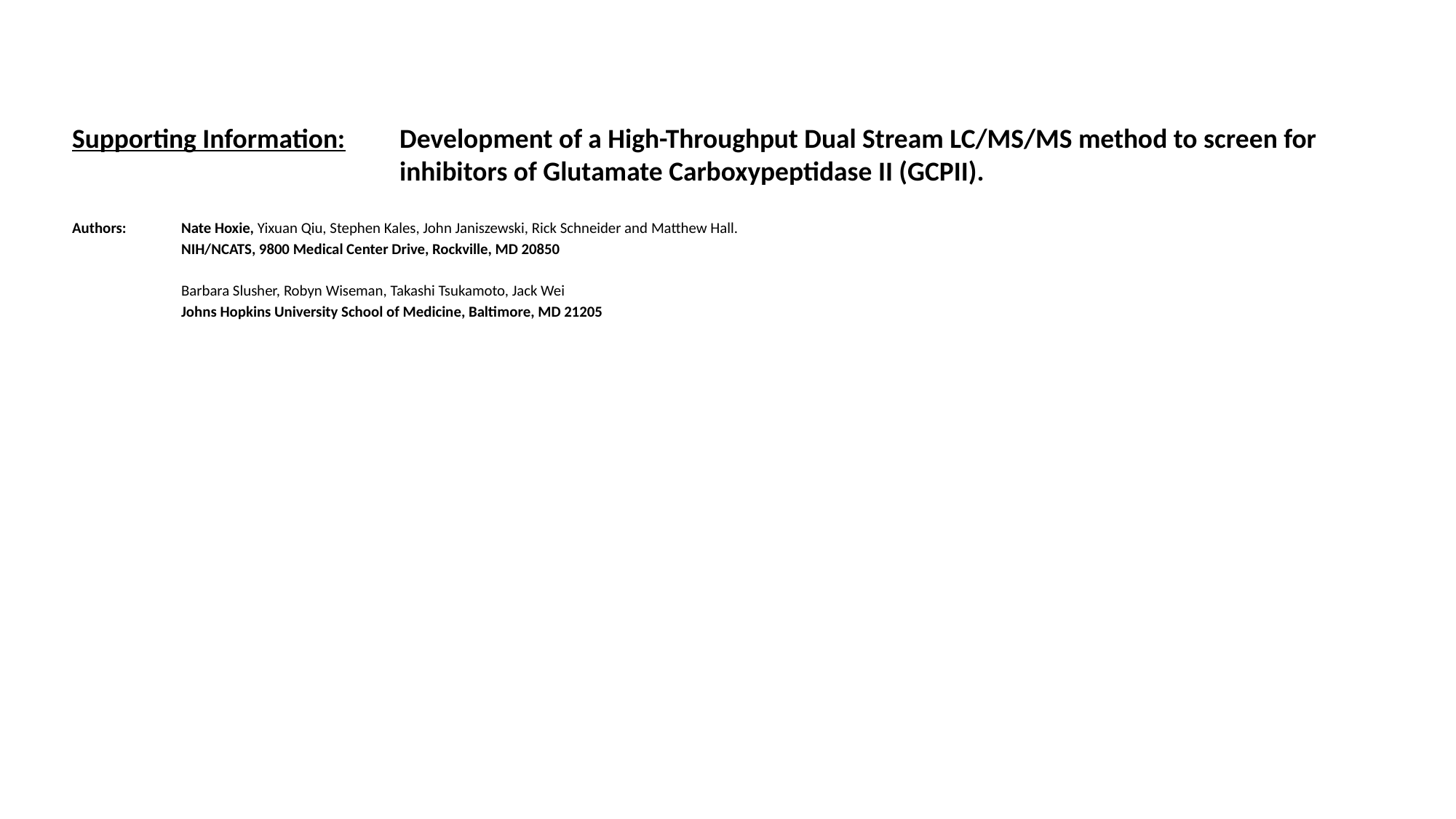

Supporting Information:	Development of a High-Throughput Dual Stream LC/MS/MS method to screen for 				inhibitors of Glutamate Carboxypeptidase II (GCPII).
Authors: 	Nate Hoxie, Yixuan Qiu, Stephen Kales, John Janiszewski, Rick Schneider and Matthew Hall.
	NIH/NCATS, 9800 Medical Center Drive, Rockville, MD 20850
Barbara Slusher, Robyn Wiseman, Takashi Tsukamoto, Jack Wei
Johns Hopkins University School of Medicine, Baltimore, MD 21205

## Slide 2
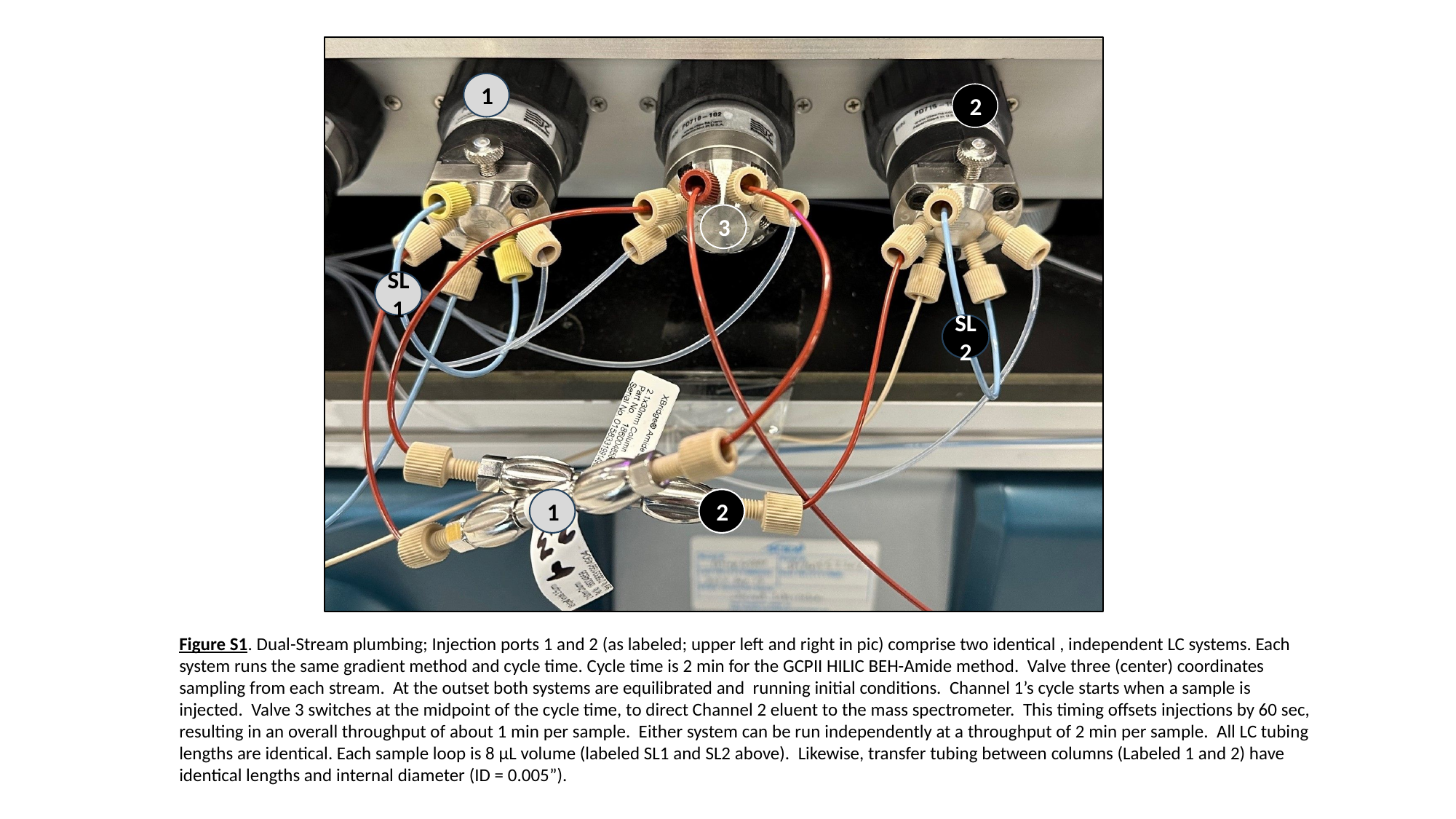

1
2
3
sl1
sl2
2
1
Figure S1. Dual-Stream plumbing; Injection ports 1 and 2 (as labeled; upper left and right in pic) comprise two identical , independent LC systems. Each system runs the same gradient method and cycle time. Cycle time is 2 min for the GCPII HILIC BEH-Amide method. Valve three (center) coordinates sampling from each stream. At the outset both systems are equilibrated and running initial conditions. Channel 1’s cycle starts when a sample is injected. Valve 3 switches at the midpoint of the cycle time, to direct Channel 2 eluent to the mass spectrometer. This timing offsets injections by 60 sec, resulting in an overall throughput of about 1 min per sample. Either system can be run independently at a throughput of 2 min per sample. All LC tubing lengths are identical. Each sample loop is 8 µL volume (labeled SL1 and SL2 above). Likewise, transfer tubing between columns (Labeled 1 and 2) have identical lengths and internal diameter (ID = 0.005”).

## Slide 3
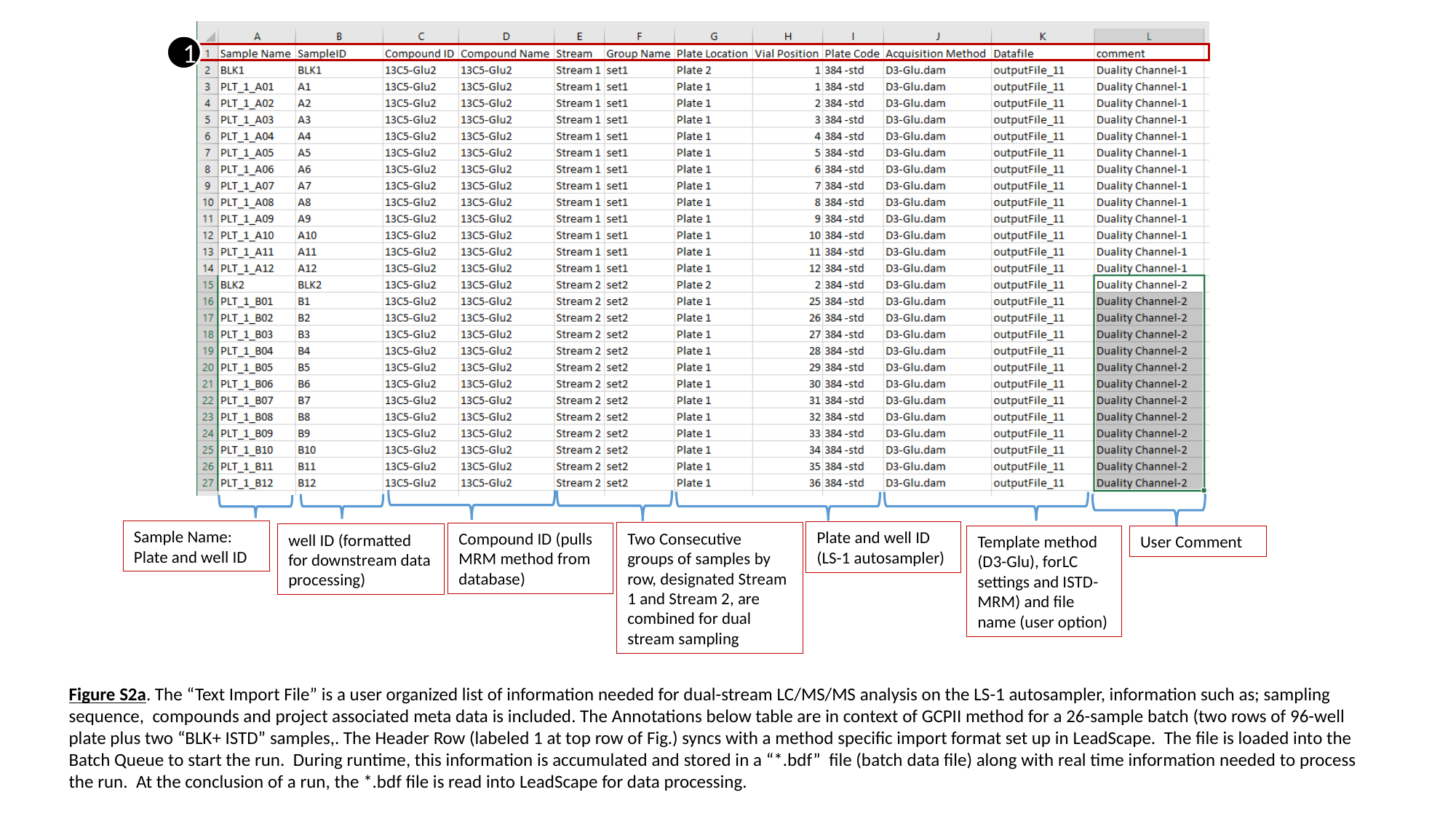

1
Sample Name: Plate and well ID
Plate and well ID (LS-1 autosampler)
Two Consecutive groups of samples by row, designated Stream 1 and Stream 2, are combined for dual stream sampling
Compound ID (pulls MRM method from database)
well ID (formatted for downstream data processing)
Template method (D3-Glu), forLC settings and ISTD-MRM) and file name (user option)
User Comment
Figure S2a. The “Text Import File” is a user organized list of information needed for dual-stream LC/MS/MS analysis on the LS-1 autosampler, information such as; sampling sequence, compounds and project associated meta data is included. The Annotations below table are in context of GCPII method for a 26-sample batch (two rows of 96-well plate plus two “BLK+ ISTD” samples,. The Header Row (labeled 1 at top row of Fig.) syncs with a method specific import format set up in LeadScape. The file is loaded into the Batch Queue to start the run. During runtime, this information is accumulated and stored in a “*.bdf” file (batch data file) along with real time information needed to process the run. At the conclusion of a run, the *.bdf file is read into LeadScape for data processing.

## Slide 4
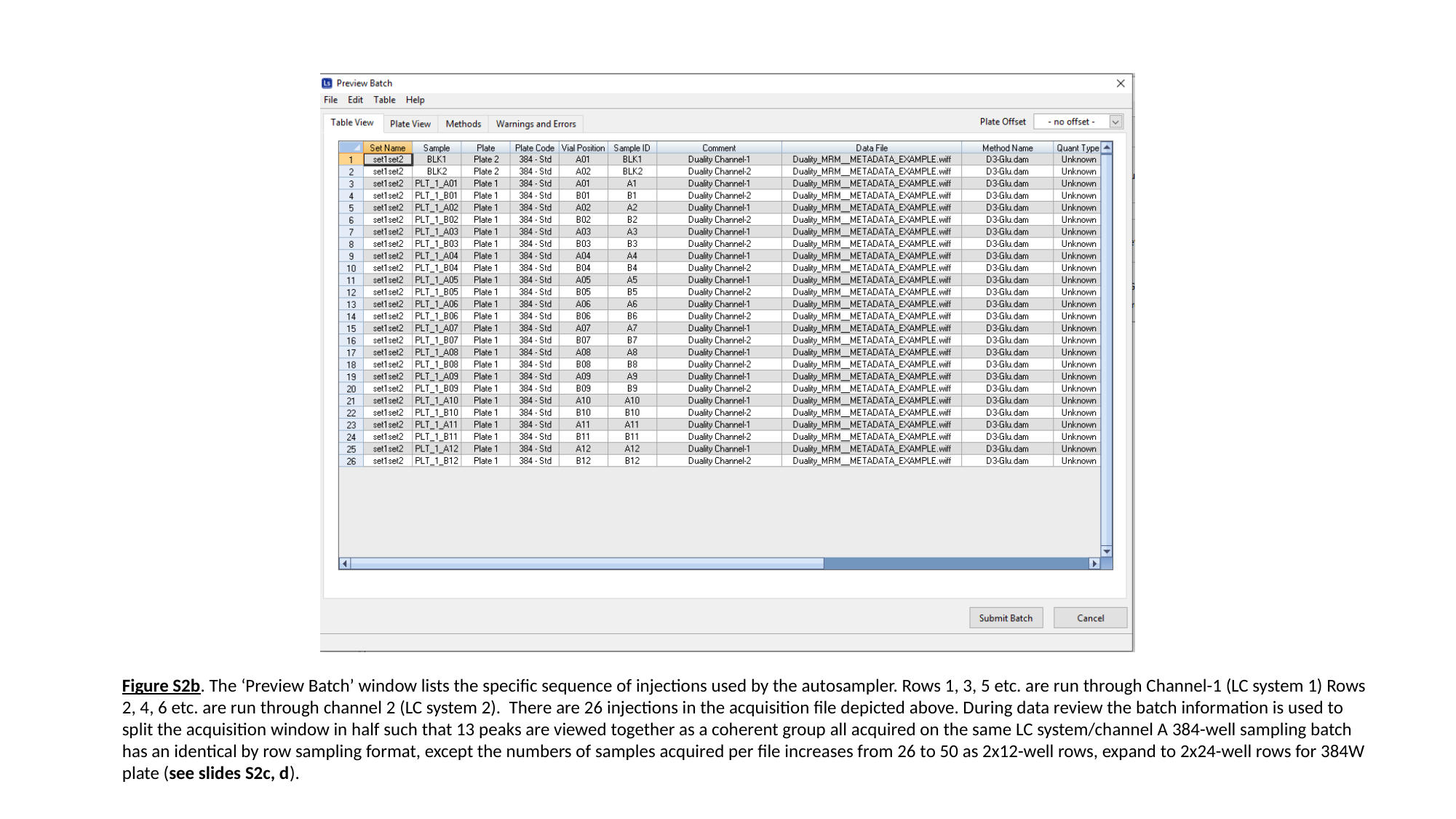

Figure S2b. The ‘Preview Batch’ window lists the specific sequence of injections used by the autosampler. Rows 1, 3, 5 etc. are run through Channel-1 (LC system 1) Rows 2, 4, 6 etc. are run through channel 2 (LC system 2). There are 26 injections in the acquisition file depicted above. During data review the batch information is used to split the acquisition window in half such that 13 peaks are viewed together as a coherent group all acquired on the same LC system/channel A 384-well sampling batch has an identical by row sampling format, except the numbers of samples acquired per file increases from 26 to 50 as 2x12-well rows, expand to 2x24-well rows for 384W plate (see slides S2c, d).

## Slide 5
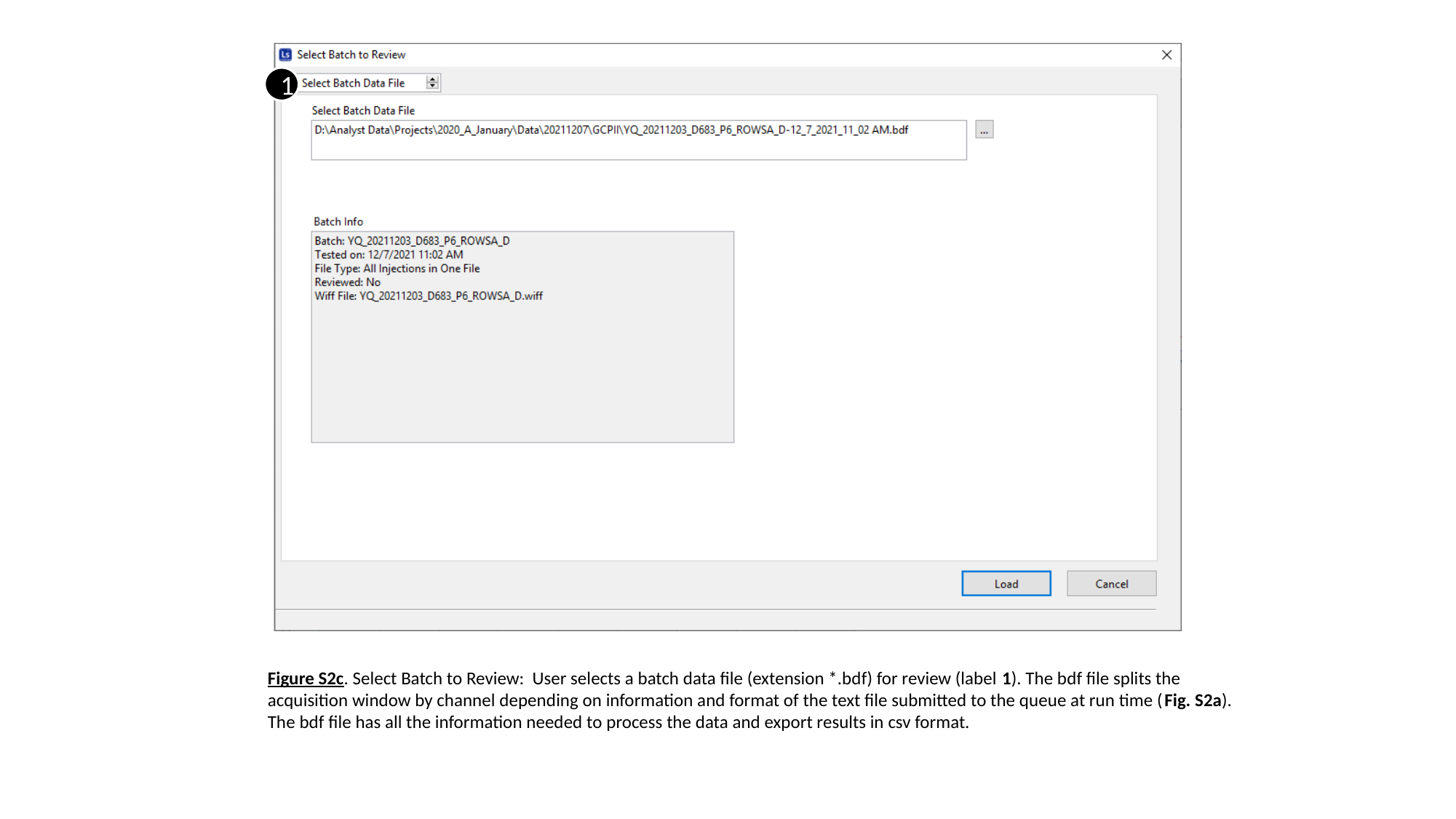

1
Figure S2c. Select Batch to Review: User selects a batch data file (extension *.bdf) for review (label 1). The bdf file splits the acquisition window by channel depending on information and format of the text file submitted to the queue at run time (Fig. S2a). The bdf file has all the information needed to process the data and export results in csv format.

## Slide 6
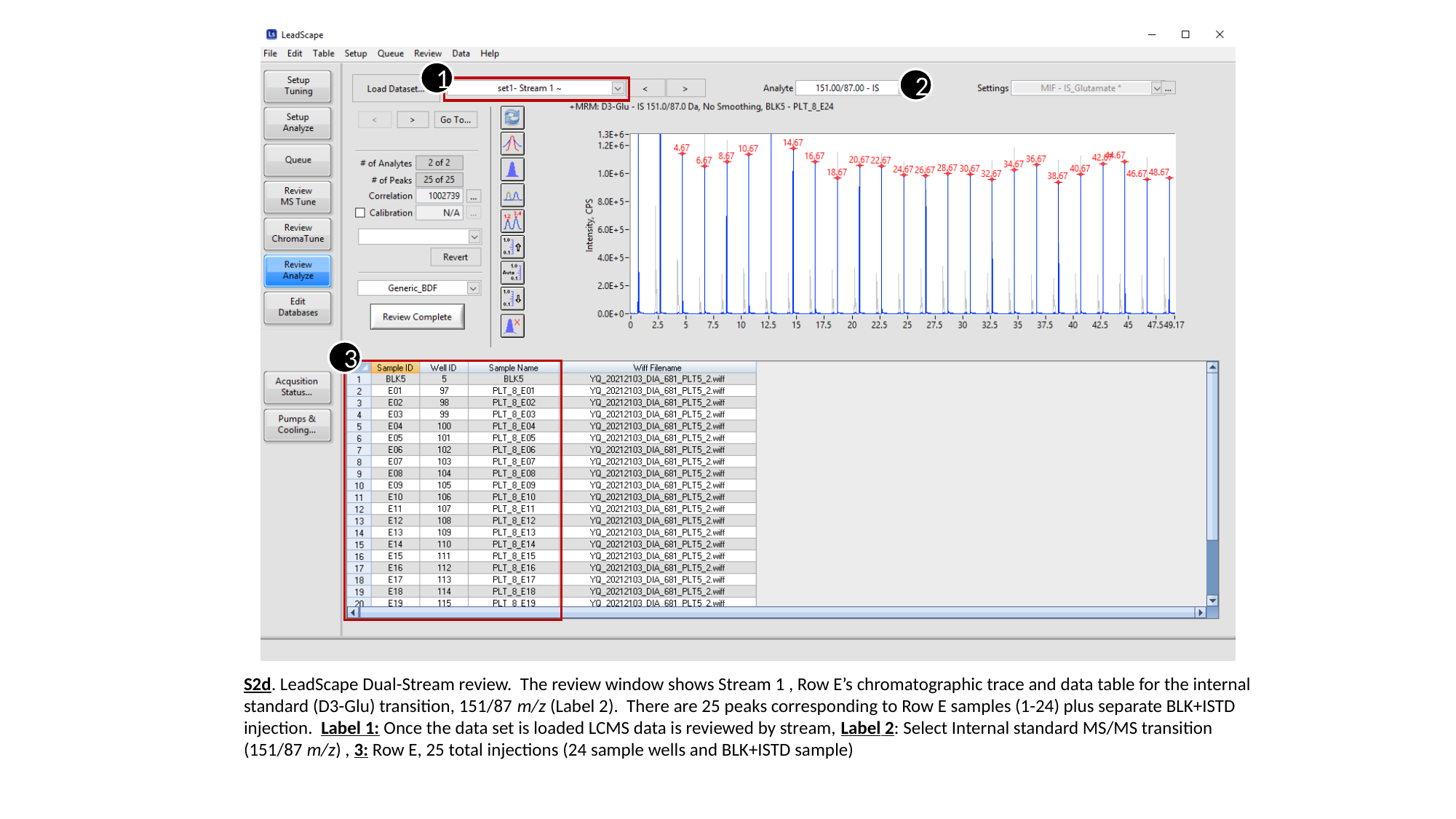

1
2
3
S2d. LeadScape Dual-Stream review. The review window shows Stream 1 , Row E’s chromatographic trace and data table for the internal standard (D3-Glu) transition, 151/87 m/z (Label 2). There are 25 peaks corresponding to Row E samples (1-24) plus separate BLK+ISTD injection. Label 1: Once the data set is loaded LCMS data is reviewed by stream, Label 2: Select Internal standard MS/MS transition (151/87 m/z) , 3: Row E, 25 total injections (24 sample wells and BLK+ISTD sample)

## Slide 7
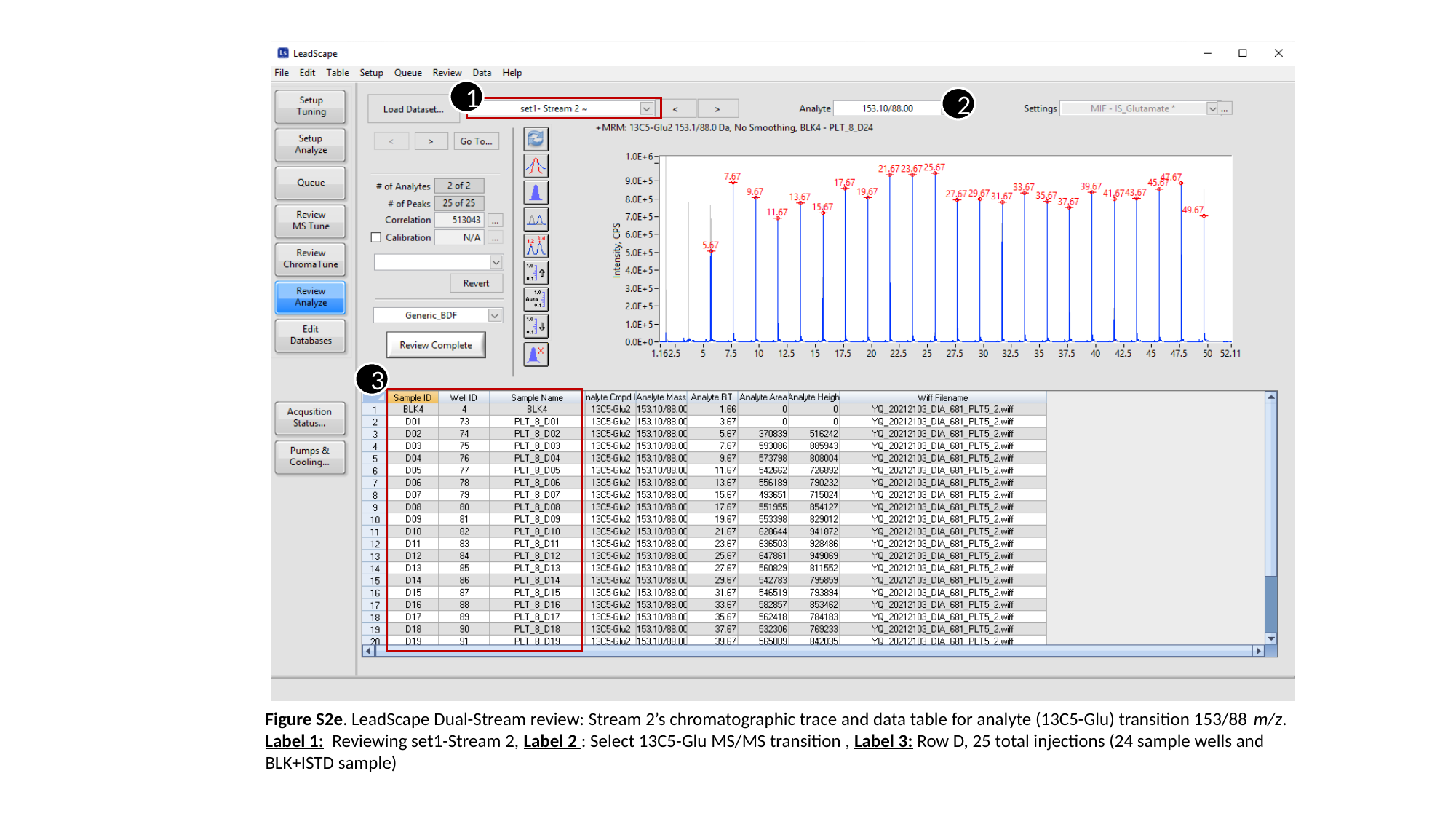

1
2
3
Figure S2e. LeadScape Dual-Stream review: Stream 2’s chromatographic trace and data table for analyte (13C5-Glu) transition 153/88 m/z. Label 1: Reviewing set1-Stream 2, Label 2 : Select 13C5-Glu MS/MS transition , Label 3: Row D, 25 total injections (24 sample wells and BLK+ISTD sample)

## Slide 8
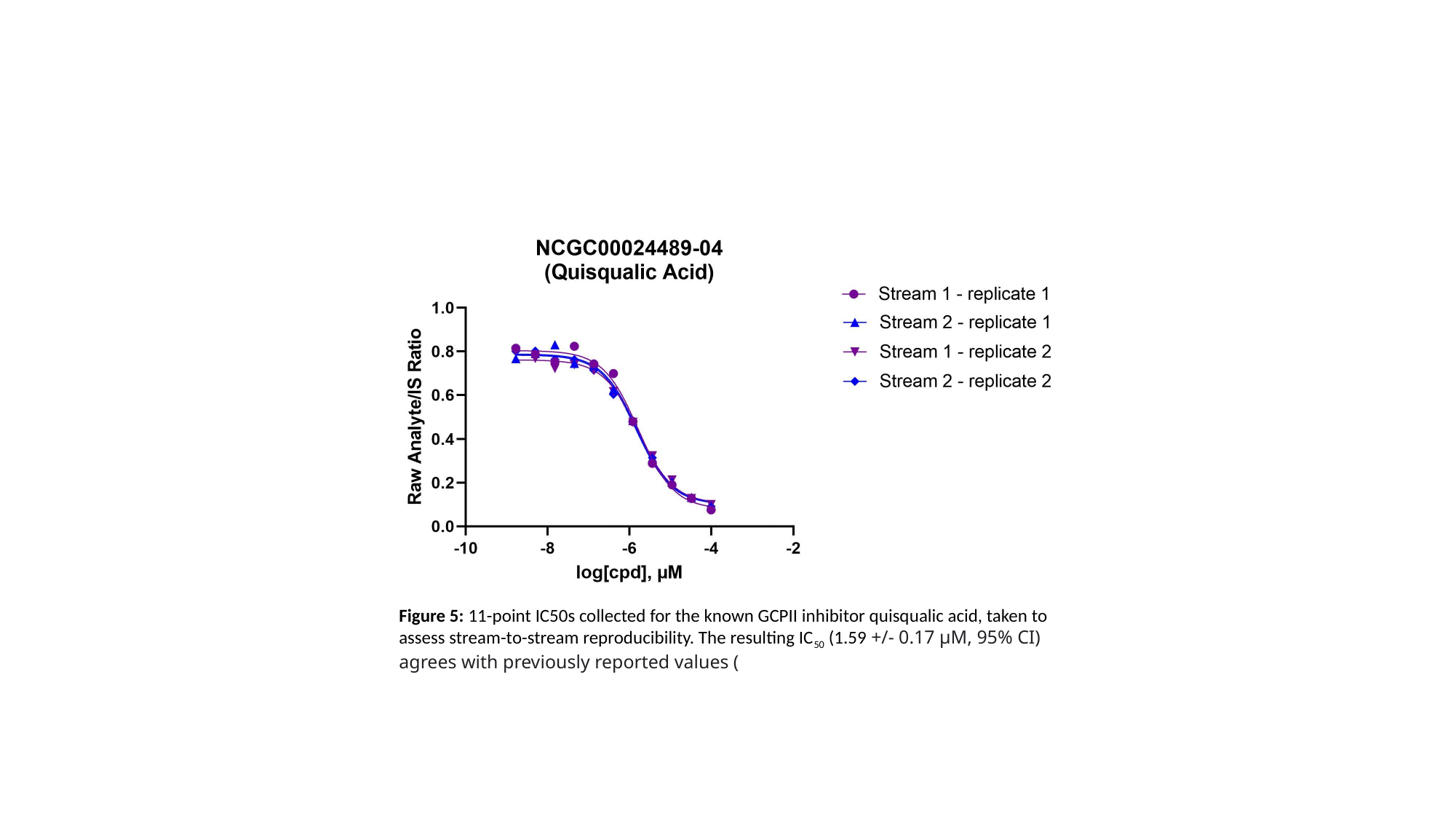

Figure 5: 11-point IC50s collected for the known GCPII inhibitor quisqualic acid, taken to assess stream-to-stream reproducibility. The resulting IC50 (1.59 +/- 0.17 µM, 95% CI) agrees with previously reported values (
